# Risk Factors for Fall-Related Mild Traumatic Brain Injuries Among Older Adults: A Systematic Review Highlighting Research Gaps

**DOI:** 10.3390/ijerph22020255

**Published:** 2025-02-11

**Authors:** Albert K. Okrah, Shafer Tharrington, Isaac Shin, Aaron Wagoner, Katelyn S. Woodsmall, Deborah A. Jehu

**Affiliations:** 1 Department of Community & Behavioral Health Sciences, School of Public Health, Augusta University, Augusta, GA 30912, USA; 2 Robert B. Greenblatt, M.D. Library, College of Allied Health Sciences, Augusta University, Augusta, GA 30901, USA

**Keywords:** brain concussion, brain injuries, head injury, accidental falls, fall risk, aged, systematic review

## Abstract

Mild traumatic brain injury (mTBI) is commonly undiagnosed, delaying treatment and recovery. Approximately 80% of mTBIs in older adults stem from falls, yet the predictive factors remain unclear. This systematic review aimed to examine the risk factors for fall-related mTBIs among older adults. The Preferred Reporting Items for Systematic Reviews and Meta-Analysis (PRISMA) protocol and the Meta-Analysis of Observational Studies in Epidemiology (MOOSE) guidelines were followed (Prospero ID: CRD42023377847). The scope included prospective studies analyzing the risk factors for fall-related mTBIs in adults ≥ 60 years. The primary outcome measure was the relative risk for fall-related mTBIs, and the secondary outcomes were fall rate, total falls, and faller/non-faller count among those with and without an mTBI. CINAHL Plus, Health Source: Nursing Academic Edition, Nursing and Allied Health Database, Medline via PubMed, SPORTDiscus, and Web of Science were searched on 4 November 2022 and 31 May 2024. Additional electronic searches were conducted. Two authors planned to screen the articles and assess the quality and risk of bias, with a third author adjudicating disagreements. Results were to be presented in a narrative synthesis. The database search yielded 434 records; 410 titles and abstracts were screened after deduplication, and 71 reports underwent a full-text review. No prospective observational studies were eligible because they did not fulfil the following: (1) focus on an mTBI (46 records); (2) exclusively assess individuals aged ≥60 (20 records); or (3) examine falls (5 records). Given the devastating consequences of fall-related mTBIs among older adults, there is an urgent need to identify the risk factors to improve screening and intervention.

## 1. Introduction

Mild traumatic brain injury (mTBI) is common among older adults [1]. An mTBI is defined as having a “Glasgow Coma Scale score between 13 and 15, 30 min after the injury, and one or more of the following symptoms: loss of consciousness <30 min; post-traumatic amnesia <24 h; impaired mental state at time of accident (confusion, disorientation, etc.); transient neurological deficit (including focal signs, epilepsy, or non-surgical intracranial injury)” [2]. There are an estimated 502,908 older adults aged 65 and above who experience an mTBI annually in the United States [3]. This number may underrepresent the true incidence, as many older adults do not seek medical attention [4]. Approximately 80.1% of all mTBIs among older adults are attributed to falls [3,5]. While most people with an mTBI recover within a year, about 20% experience persistent symptomatic, cognitive, emotional, behavioral, or physical impairments [6,7,8]. Mortality rates are significantly elevated among older adults who experience an mTBI compared to young adults [9], yet the majority of research has been focused on mTBIs in sports or the military rather than mTBIs among older adults [10,11]. Neglecting to include older adults in fall-related mTBI research results in critical gaps in care delivery, including delayed diagnosis and a lack of age-specific rehabilitation strategies—ultimately contributing to adverse outcomes, such as prolonged recovery, increased functional decline, and higher mortality rates [12,13]. Therefore, mitigating the cognitive, psychosocial, and physical limitations caused by fall-related mTBIs in older adults should be a frontline priority.

Several fall risk factors may contribute to a fall-related mTBI among older adults, spanning domains including sociodemographic (e.g., age, sex), balance and mobility (e.g., slow gait, postural instability), sensory and neuromuscular (e.g., vision impairment, proprioceptive deficits), psychological (e.g., depression, anxiety), medical (e.g., osteoporosis, Parkinson’s disease), medication (e.g., polypharmacy, sedatives), and environmental (e.g., clutter, dim lighting) [14]. It is possible that one or a combination of these fall risk domains are associated with a greater risk for fall-related mTBI than others [15]. For example, older adults with an mTBI may present with lower mood, poorer cognition, anxiety, as well as vestibular and ocular–motor dysfunction compared to older adults without an mTBI [16]. However, to our knowledge, no systematic review has examined the risk factors for fall-related mTBIs among older adults [17].

The purpose of our systematic review was to examine the risk factors associated with fall-related mTBIs in older adults. A better understanding of the risk factors for fall-related mTBIs may inform screening and monitoring strategies for older adults and help to prevent future fall-related mTBIs.

## 2. Materials and Methods

### 2.1. Study Design

This systematic review was reported according to the Preferred Reporting Items for Systematic Reviews and Meta-Analysis (PRISMA) protocol and the Meta-Analysis of Observational Studies in Epidemiology (MOOSE) guidelines [18,19]. The study was registered a priori on the International Prospective Register of Systematic Reviews (PROSPERO) database (Prospero ID: CRD42023377847); we did not publish the protocol in an academic journal. The results were presented as a conference poster [20].

### 2.2. Eligibility Criteria

#### 2.2.1. Inclusion Criteria

The eligibility criteria for the articles were as follows: (1) focused on mTBIs; (2) involved individuals 60 years and over; (3) investigated falls; (4) were prospective quantitative studies; and (5) were published in English. The inclusion was limited to studies with a prospective 12-month fall monitoring period to assess baseline risk factors influencing future fall-related mTBIs [21,22]. The decision to include only prospective studies was based on guidelines suggesting that prospective fall risk assessments are more reliable, as they avoid recall bias and the secondary complications of falls that can affect retrospective analyses [23,24]. All the eligible articles were planned to be included to obtain the maximum amount of information, even those with multiple publications. To maintain a structured approach in assessing the articles against our initial eligibility criteria, a hierarchical review process was applied. Only peer-reviewed studies were included in the final analysis; this additional search helped us to reduce publication bias by identifying sources outside the standard databases, providing a more comprehensive search of the topic [25,26].

#### 2.2.2. Exclusion Criteria

The exclusion criterion, as outlined in our published protocol, was to exclude studies that only included retrospective fall data. More specifically, an mTBI may alter the fall risk profile of an individual by provoking symptoms (e.g., headache, dizziness, nausea, and fogginess) and/or changes in cognitive, psychological, and physical function [27]. Consequently, retrospectively assessing modifiable fall risk factors to predict a fall-related mTBI after it occurred could lead to inaccurate conclusions about whether these factors are a consequence or cause of the mTBI.

### 2.3. Population, Exposure, Outcome (PEO)

The PEO framework [28] guided the formulation of search strategies used to determine the risk factors for fall-related mild traumatic brain injuries among older adults as outlined below:

Population: Older adults aged 60 and older;

Exposure: Falls;

Outcomes: The primary outcome of the study was the relative risk for a fall-related mTBI depending on the fall risk domain (sociodemographic, balance and mobility, sensory and neuromuscular, psychological, medical, medication use, and environmental). Secondary outcomes of interest were the fall rate (falls/year), the total number of falls (count), and the total number of fallers and non-fallers in those with and without an mTBI (count).

### 2.4. Electronic Database Searches

The following electronic databases were searched: CINAHL Plus (1985–2024), Health Source: Nursing/Academic Edition (1946–2024), Nursing and Allied Health Database (1857–2024), Medline via PubMed (1879–2024), SPORTDiscus with Full Text (1573–2024), and Web of Science (1977–2024). The initial search took place on 4 November 2022 (File S1), followed by a second search on 31 May 2024 (File S2). Both searches were conducted by the same librarian (ST). The search strategies included a combination of key concepts in aging, falls, and head injuries with Boolean operators. In the case of missing data, we planned to contact the primary authors to determine whether the results were available and then included these studies in the systematic review if they were appropriate. The search results were exported to EndNote where one author removed any duplicates (ST). Three authors screened the search results against the eligibility criteria (IGS, ADW, AKO). If disagreements occurred among the three authors that were not resolved, a fourth member became involved to settle the disagreements (KSW). A fifth reviewer was used when necessary (DAJ). MeSH terms and database-specific keywords were used because they have been shown to enhance the searches’ ability to retrieve the relevant citations (sensitivity) and distinguish between the relevant and non-relevant results (specificity) [29,30]. A full list of the MeSH and keyword search terms for each database is provided in Files S1 and S2.

Additional electronic searches were conducted on 4 November 2022, by examining reference lists of the relevant studies (IGS, ADW, KSW) and consulting content experts (DAJ) and, again on 31 May 2024, by manually reviewing the articles of key authors (AKO, DAJ) and consulting experts (DAJ).

### 2.5. Data Extraction

The data were set to be extracted using a data extraction sheet that consisted of the following categories: Sociodemographic Factors, Balance and Mobility Factors, Sensory and Neuromuscular Factors, Psychological Factors, Medical Factors, Medications, and Environmental Factors. The data extraction sheets for assessing the primary and the secondary outcome measures are provided in Files S3 and S4, respectively. Three authors were designated to extract the data (IGS, ADW, KSW). A fall was defined as “an unexpected event in which the participant comes to rest on the ground, floor, or lower level” [22]. Risk was defined as “the probability that an unwanted health event (e.g., a future fall) will occur” [31].

### 2.6. Quality of Reporting and Risk of Bias

Two authors (IGS, ADW) were designated to assess the reporting quality of the included articles using the Strengthening the Reporting of Observational studies in Epidemiology (STROBE) checklist, which has shown good reliability [32]. Adherence to the STROBE checklist has been noted to greatly enhance the quality and transparency of observational research, an important ethical obligation in publication [33]. A study would have been deemed high quality if ‘yes’ was selected for each of the 22 items of the STROBE checklist for cohort and case-control prospective studies. Conversely, low quality would have been assigned if one or more ‘no’ responses were selected.

Two independent reviewers (IGS, ADW) planned to assess the risk of bias in each study using the Joanna Briggs Institute Prevalence Critical Appraisal (JBI-PCA) tool and compared their results for discrepancies. This tool was developed to assess the methodological quality of studies reporting prevalence data to be included in systematic reviews [34]. A low risk of bias would have been assigned to each eligible study if “yes” was selected for each of the nine questions. In cases of discrepancies between reviewers, disagreements were planned to be resolved through discussion with a third neutral reviewer (DAJ).

The certainty of evidence was intended to be assessed using the Grading of Recommendations Assessment, Development, and Evaluation (GRADE) approach [35]. Tabulating the summary findings according to the GRADE guidelines is recognized as an effective method for illustrating the clinical implications of review results [36,37].

### 2.7. Data Synthesis

The Cochrane Effective Practice and Organization of Care (EPOC) guidelines for reporting empty reviews [38] were followed, and the number of excluded studies was indicated, as recommended [39]. Where appropriate, the findings would have been presented in a narrative synthesis [40], structured around the primary fall risk outcome measures, with data displayed in tables and figures. Descriptive statistics would have been used to summarize the characteristics of the included studies, such as citation, sample size, percentage of female participants, mean age, and the duration of prospective falls during follow-up.

## 3. Results

### 3.1. Study Selection

The initial search strategy resulted in a total of 410 results. After eliminating n = 23 duplicates, 387 underwent title and abstract screening. A total of 63 articles underwent full-text review. The second search strategy resulted in a total of 24 results. After removing 1 duplicate, 23 titles and abstracts were screened, leading to 8 articles undergoing full-text review.

### 3.2. Included Studies

**Figure 1:**
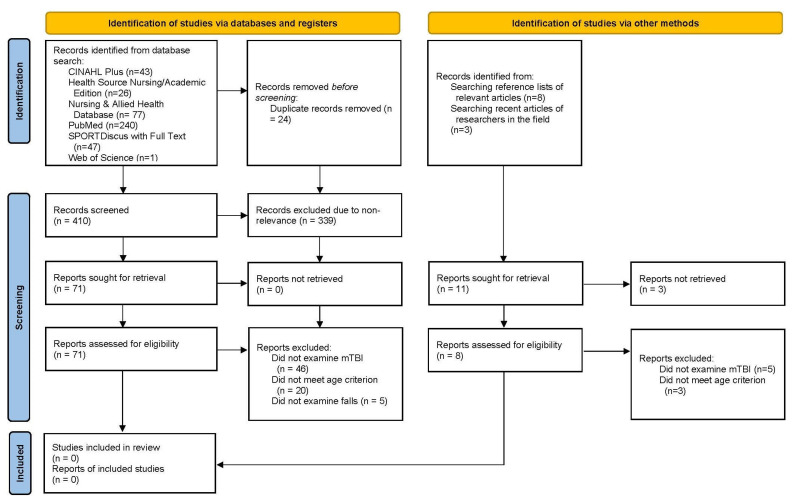
PRISMA flow diagram of the identification, screening, and eligibility of records and reports.

No studies were found that met the study eligibility criteria for this review (Figure 1). The lack of included studies highlights a significant gap in the literature regarding the predictors of fall-related mTBIs among older adults. Since no studies met the inclusion criteria, the STROBE checklist, JBI-PCA tool, and the GRADE guidelines were not utilized in this systematic review.

### 3.3. Excluded Studies

Out of the 71 studies assessed from the database search, a total of 46 were excluded because they did not examine the mTBI, 20 did not meet the age requirement and 5 did not examine falls. Additionally, five studies identified from other electronic searches were excluded for not examining mTBI and three for not meeting the age requirement. Many studies were excluded for multiple reasons, but each was counted only once for a specific reason. No study was excluded solely because it was not a prospective study.

## 4. Discussion

No prospective studies were found that examined our primary outcome measure (the relative risk for fall-related mTBIs in older adults) or secondary outcome measures (fall rate, total number of falls, and the total number of fallers and non-fallers in those with and without an mTBI). The current cross-sectional data on ground-level fall-related mTBIs among older adults cover only two fall-risk factor domains, including sociodemographic (e.g., alcohol ingestion [41,42]) and environmental risk factors (e.g., falling from stairs compared to falling over [43,44] type of facility—commercial compared to residential, outdoor location [43], floor conditions—non-slippery, concrete, and sloped flooring [45,46], and the presence of obstacles [43]). Notably, no specific data exist for other important domains including balance and mobility, psychological, medical, medication, as well as sensory and neuromuscular factors. It is also important to acknowledge that research on fall predictors using retrospective analyses may be less accurate due to recall bias and secondary complications from falls [21]. Thus, researchers recommend fall tracking over a 12-month prospective period for more precise data [23].

Publishing an empty systematic review, defined as a review that identifies no studies meeting its predefined eligibility criteria [38], is valuable for highlighting major research gaps and informing researchers, stakeholders, and other decision-makers about the lack of robust evidence [47]. Empty reviews can also influence clinical practice guidelines, as demonstrated in the two clinical practice guidelines [48,49] developed in response to an empty systematic review [50]. There are several barriers contributing to the lack of research on fall-related mTBIs in older adults, including issues related to researchers, funding sources, healthcare providers, screening tools, and patients.

### 4.1. Reasons for a Lack of Research on Fall-Related mTBI

Researchers contribute to the low number of studies on fall-related mTBIs in older adults. One significant issue is the frequent practice of collapsing an mTBI and a TBI (traumatic brain injury) into a single category [51], which complicates the ability to distinguish between the two conditions and their respective impacts. Other studies employed retrospective or cross-sectional designs, which are prone to fall recall bias compared with the gold standard prospective fall monitoring [52,53]. Some studies also include a wide age range, despite the varying mechanism of injury and prevalence across the lifespan [54,55]. Most previous clinical trials on head injuries have excluded individuals over the age of 65 years [56], despite the strict exclusion criteria such as psychiatric and neurological conditions [57]. Moreover, the absence of research on fall-related mTBIs in older adults points to a possible lack of funding prioritization for this critical area.

Issues in clinical practice may also contribute to the identified research gap. Many mTBI diagnoses are overlooked by clinicians. One major reason is the lack of education and training on mTBIs in medical schools and emergency departments [58,59]. Additionally, some clinicians perceive mTBIs primarily as high-impact injuries (e.g., sports-related, motor vehicle collision), and this assumption impacts the care of injuries outside the sports context [60,61]. When the fall results in multiple injuries, clinicians and staff at residential care facilities often prioritize what they consider to be more severe injuries (e.g., broken bones) over mTBIs or do not allocate sufficient time to evaluate head injuries [17,62]. In the absence of bleeding or loss of consciousness, some clinicians and residential care facility staff may diminish the severity of the head injury and not recommend further treatment [17]. There are also reports of clinicians neglecting to document mTBI cases in clinical records or improperly classifying head injuries, resulting in gaps in care [63,64].

In cases where the healthcare providers suspect an mTBI, diagnosis may be difficult because the existing technology is too limited to provide accurate detection [64]. Current widely used assessment tools, such as the Sports Concussion Assessment Tool (SCAT 5) [65], were developed for a younger mTBI population and are limited to sport contexts. For example, questions such as “Who scored last in this match?” are not relevant to older adults [66]. Another widely used assessment tool for evaluating people with head injuries, the Glasgow Coma Scale [67], has been implicated by some studies as a poor differentiator between the levels of head injury severity [68]. These screening tools are designed to detect injuries from high-energy mechanisms [69], which are less applicable to older adults, who often experience low-energy falls [70]. Additionally, the outcome assessments typically used for more severely injured patients lack the sensitivity needed to assess the subtle cognitive and behavioral sequelae that often result from an mTBI [71]. Studies indicate that computed tomography (CT) is not sensitive enough to detect mTBIs in older adults presenting to emergency departments [72], and magnetic resonance imaging (MRI) is often too expensive and impractical for the regular assessment of mTBI in the acute phase [72].

Underreporting and unawareness of mTBIs among older adults also contribute to the low number of studies on this condition. A national survey of Medicare beneficiaries found that less than 50% of the older adults who fell the previous year informed their healthcare provider of the event [73]. Other studies have reported that many older adults are afraid that their independence will be taken away if they admit to falling, leading to the underreporting of falls [74,75]. Additionally, some older adults may be unaware of their mTBI because the symptoms are subtle and unalarming, often becoming apparent only during symptom-provoking exertional exercise [76,77]. Furthermore, the presence of more acute injuries takes priority [78], leading older adults to ignore the mTBI in favor of addressing other injuries. Given that prospective studies may rely on medical records for identifying potential participants, underreported or misreported cases of mTBI could significantly hinder recruitment efforts, thereby impacting the lack of research in this area.

In conclusion, the diagnosis and management of mTBIs in older adults is a complex issue that is often overlooked due to a multitude of factors. These include a lack of research focus and funding for this population, a lack of education and training among physicians, limitations in current diagnostic technology, misconceptions about the severity of the head injury, and underreporting and unawareness among older adults themselves. Addressing these issues through enhanced education, the development of more sensitive diagnostic tools, increased awareness, and targeted research are essential to better understand risk factors for fall-related mTBIs and ensure better care and outcomes for older adults with an mTBI.

### 4.2. Implications

This systematic review has several implications. The accurate diagnosis of an mTBI is critical for ensuring that older adults who present to clinics with an mTBI are appropriately managed [79]. Therefore, this lack of research implies that older adults with an mTBI may be undiagnosed or misdiagnosed, leading to some patients receiving conflicting treatments or no treatment at all [79]. This oversight poses a substantial risk to older adults, who may suffer enduring consequences, such as an increased risk of dementia, even from a single incidence of fall-related head injury [80,81]. Older adults also tend to develop concerns about falling post-mTBI and often adopt maladaptive strategies like reduced physical activity and social isolation, which may further increase the risk of future falls [82,83,84]. This systematic review highlights the need for cohort and case-control prospective studies to examine multiple fall risk domains in determining the risk factors for fall-related mTBIs.

### 4.3. Strengths and Limitations

A limitation of this systematic review is that the database search only included studies published in the English language. Acknowledging the possibility of missing relevant articles using the search strategy employed, a thorough database search across six databases was conducted with the assistance of a librarian, and additional electronic searches were also performed. Additionally, although there was a plan to search Google Scholar for citations of eligible articles, no studies met our inclusion criteria; thus, our additional electronic search focused on expert consultation and the manual review of relevant studies and authors. The strengths of this systematic review include our efforts to minimize bias by involving two independent authors in the review process. The target population was not narrowed, and older adults in the community and residential care were intentionally included. Publishing an empty review emphasizes the need for further investigation, calls stakeholders to address gaps, and encourages researchers to focus on this critical issue.

## 5. Conclusions

The risk factors for fall-related mTBIs in older adults remain unknown, as no prospective studies met our eligibility criteria. This lack of research highlights a critical gap in the field. The lack of eligible studies could be a result of insufficient research prioritization by researchers, funders, inadequate training and awareness among healthcare professionals, and limited knowledge and proactive engagement of the older adults themselves. Researchers need to prioritize studying mTBIs in older adults through well-designed prospective studies, addressing methodological limitations and distinguishing mTBIs from TBIs to enhance understanding and improve outcomes. Healthcare professionals must focus on developing effective strategies for diagnosing, documenting, and monitoring mTBIs to facilitate screening and timely therapeutic interventions. Older adults should be more aware of the signs and risks of mTBIs and seek prompt medical attention to ensure proper diagnosis and management.

## Data Availability

The original contributions presented in the study are included in the article/Supplementary Material, further inquiries can be directed to the corresponding author.

## Supplementary Materials

The following supporting information can be downloaded at: https://www.mdpi.com/article/10.3390/ijerph22020255/s1, File S1—Initial electronic search results, File S2—Second electronic search results, File S3—Assessment sheet for relative risk for fall-related mTBIs and File S4—Study Details of Falls resulting in mTBI studies.
