# Supplementary material for: Risk Factors for Fall-Related Mild Traumatic Brain Injuries Among Older Adults: A Systematic Review Highlighting Research Gaps
Source: Int J Environ Res Public Health. 2025 Feb 11;22(2):255. doi: 10.3390/ijerph22020255 (PMC11854998; doi:10.3390/ijerph22020255)
Supplement: Supplementary file 1 [file ijerph-22-00255-s001.zip › ijerph-3410783- Files S1 and S2 - Electronic search results.pdf]

International Journal of
Environmental Research and Public Health

MDPI

File S1

Initial Database Search Strategy

Search Date: 11/3/22
Before De-duplication
CINAHL Plus: 39
Health Source: Nursing/Academic Edition: 21
Nursing & Allied Health Database: 77
Medline via PubMed: 225
SPORTDiscus with Full Text: 47
Web of Science: 1
Citation Total: 410

After De-Duplication
CINAHL Plus: 37
Health Source: Nursing/Academic Edition: 19
Nursing & Allied Health Database: 70
Medline via PubMed: 219
SPORTDiscus with Full Text: 41
Web of Science: 1
Citation Total: 387

CINAHL Plus (1985-2022)

Line	Search Term	Hits
S1	(MH “Aging”) OR (MH “Aged, 80 and Over”) OR (MH “Age Factors”) OR (MH “Geriatricians”) OR (MH “Frailty Syndrome”)	490,467
S2	elder* OR geriatric* OR ((old* or aged) N1 (person* OR adult* OR people OR patient*)) OR frail OR senior*	338,192
S3	S1 OR S2	708,944
S4	(MH “Head Injuries”) OR (MH “Contusions and Abrasions”) OR (MH “Brain Injuries”) OR (MH “Brain Concussion”) OR (MH “Brain Contusions”)	40,712
S5	Contusion OR concussion OR head injury OR mTBI OR “mild traumatic brain injury”	27,757
S6	S4 OR S5	50,380
S7	(MH “Fall Risk Assessment Tool”) OR (MH “Risk Assessment”) OR (MH “Risk Factors”)	314,306
S8	Fall risk	11,355
S9	S7 OR S8	322,272
S10	(MH “Accidental Falls”)	25,822
S11	Fall*	81,058
S12	S10 OR S11	81,058
S13	MH “Prospective Studies”	512,638
S14	(prospective or follow up)	779,150
S15	S13 OR S14	779,150
S16	S3 AND S6 AND S9 AND S12 AND S15	39
S17	Filter for English	39

Health Source: Nursing/Academic Edition (1946-2022)

Line	Search Term	Hits
S1	XX "aging" OR XX "geriatrician" OR XX "geriatric" OR XX "adult"	150,994
S2	TX “aged 80 and over” OR TX “frailty syndrome” OR TX “age factors” OR TX “frail” OR TX “elderly” OR TX “aged” OR TX “older” OR TX “elder” OR TX “geriatric” OR TX “elderly people” OR TX “old people” OR TX “senior”	543,630
S3	S1 OR S2	623,354
S4	XX “contusions” OR XX “abrasion” OR XX “concussion” OR XX “TBI”	5,928
S5	TX “head injury” OR TX “head injuries” OR TX “brain injuries” OR TX “brain injury” OR TX “brain injuries” OR TX “mTBI” OR TX “mild traumatic brain injury”	26,844
S6	S4 OR S5	28,301
S7	XX “risk factors”	109,594
S8	TX “fall risk” OR TX “fall risk assessment tool” OR TX “risk assessment”	48,021
S9	S7 OR S8	145,263
S10	XX “accidental injury”	177
S11	TX “accidental falls” OR TX “fall”	116,198
S12	S10 OR S11	116,358
S13	DE "longitudinal method"	43,797
S14	S3 AND S6	11,331
S15	S14 AND S9	1,524
S16	S15 AND S12	704
S17	S16 AND S13	21
S18	Filter for English	21

International Journal of
Environmental Research and Public Health

MDPI

Nursing and Allied Health Database (1857-2022)

Line	Search Term	Hits
S1	mesh.Exact("Aging" OR "geriatrician" OR "geriatric" OR "adult")	224,651
S2	"aged 80 and over" OR "frailty syndrome" OR "age factors" OR "frail" OR "elderly" OR "aged" OR "older" OR "elder" OR "geriatric" OR "elderly people" OR "old people" OR "senior"	1,582,534
S3	S1 OR S2	1,637,273
S4	mesh.Exact("contusions" OR "abrasion" OR "concussion" OR "TBI")	165
S5	"head injury" OR "head injuries" OR "brain injuries" OR "brain injury" OR "brain injuries" OR "mTBI" OR "mild traumatic brain injury"	89,737
S6	S4 OR S5	89,869
S7	mesh.Exact("risk factors")	54,880
S8	"fall risk" OR "fall risk assessment tool" OR "risk assessment"	170,591
S9	S7 OR S8	215,303
S10	mesh.Exact("accidental falls")	2,227
S11	"accidental falls" OR "fall"	359,468
S12	S10 OR S11	359,468
S13	mesh.Exact("prospective studies" OR "Follow-Up Studies")	62,533
S14	S3 AND S6	48,894
S15	S14 AND S9	4,497
S16	S15 AND S12	1,996
S17	S16 AND S13	77
S18	Filter for English	77

International Journal of
Environmental Research and Public Health

MDPI

Medline via PubMed (1879-2022)

Line	Search Term	Hits
S1	"Aged"[Mesh] OR "Aged, 80 and over"[Mesh] OR "Aging, Premature"[Mesh] OR "Healthy Aging"[Mesh] OR "Aging"[Mesh] OR "Cognitive Aging"[Mesh] OR "Geriatrics"[Mesh] OR "Frail Elderly"[Mesh] OR "Long-Term Care"[Mesh] OR "Homes for the Aged"[Mesh]	3,643,246
S2	"geriatric care" OR "elder*" OR "old" OR "older" OR "geriatric*" OR "age" OR "aged" OR "people*" OR "patient*" OR "frail*"	12,492,132
S3	S1 OR S2	12,569,239
S4	"Head Injuries, Closed"[Mesh] OR "Head Injuries, Penetrating"[Mesh] OR "Brain Contusion"[Mesh] OR "Brain Injuries"[Mesh] OR "Brain Concussion"[Mesh]	83,190
S5	"abrasions" OR "concussion" OR "head injury" OR "mTBI"	36,971
S6	S4 OR S5	100,565
S7	"Risk Factors"[Mesh] OR "Accident Prevention"[Mesh] OR "Uncertainty"[Mesh] OR "Risk Assessment"[Mesh] OR "Geriatric Assessment"[Mesh]	1,272,069
S8	"prevention*" OR "risk*" OR "protective*" OR "assess*" OR "factor*"	11,832,800
S9	S7 OR S8	11,871,240
S10	"Accidental Falls"[Mesh]	27,654
S11	"fall*"	310,344
S12	S10 OR S11	310,344
S13	"Prospective Studies"[Mesh]	641,892
S14	"Follow-Up Studies"[Mesh]	688,076
S15	S13 OR S14	1,237,170
S16	S3 AND S6	59,137
S17	S16 AND S9	33,362
S18	S17 AND S12	1,767
S19	S18 AND S15	238
S20	Filter for English	225

International Journal of
Environmental Research and Public Health

MDPI

SPORTDiscus with Full Text (1573-2022)

Line	Search Term	Hits
S1	(DE "AGING") OR (DE "GERIATRICS")	7,880
S2	TX (“aged 80 and over” OR “adult” OR “frailty syndrome” OR “age factors” OR “frail” OR “elderly” OR “aged” OR “older” OR “elder” OR “geriatrician” OR “geriatric” OR “elderly people” OR “old people” OR “senior”)	381,434
S3	S1 OR S2	384,013
S4	(DE "BRUISES") OR (DE "BRAIN concussion") OR (DE "BRAIN injuries")	6,737
S5	TX (“contusion” OR “abrasion” OR “head injury” OR “head injuries” OR “brain injuries” OR “brain injury” OR “brain injuries” OR “mTBI” OR “mild traumatic brain injury”)	24,537
S6	S4 OR S5	26,124
S7	DE "INJURY risk factors"	1,928
S8	TX (“fall risk” OR “fall risk assessment tool” OR “risk assessment”)	17,206
S9	S7 OR S8	18,953
S10	DE "ACCIDENTAL falls"	1,865
S11	TX (“accidental falls” OR “fall”)	135,647
S12	S10 OR S11	135,647
S13	TX (“prospective studies” OR “Follow-Up Studies”)	11,327
S14	S3 AND S6	10,444
S15	S14 AND S9	620
S16	S15 AND S12	336
S17	S16 AND S13	47
S18	Filter for English	47

International Journal of
Environmental Research and Public Health

MDPI

Web of Science (Web of Science Citation Index Expanded, 1977-2022)

Line	Search Term	Hits
S1	ALL=("aging" OR "geriatrics" OR "aged 80 and over" OR "adult" OR "frailty syndrome" OR "age factors" OR "frail" OR "elderly" OR "aged" OR "older" OR "elder" OR "geriatrician" OR "geriatric" OR "elderly people" OR "old people" OR "senior")	2,594,367
S2	ALL=("Head Injuries, Closed" OR "Head Injuries, Penetrating" OR "Brain Contusion" OR "Brain Injuries" OR "Brain Concussion" OR "bruises" OR "contusion" OR "abrasion" OR "head injury" OR "head injuries" OR "brain injuries" OR "brain injury" OR "brain injuries" OR "mTBI" OR "mild traumatic brain injury")	163,681
S3	ALL=("risk factors" OR "fall risk" OR "fall risk assessment tool" OR "risk assessment")	863,415
S4	ALL=("accidental falls" OR "fall")	197,058
S5	ALL=("prospective studies" OR "follow-up studies")	52,641
S6	S1 AND S2	25,762
S7	S6 AND S3	1,797
S8	S7 AND S4	151
S9	S8 AND S5	1
S10	Filter for English	1

International Journal of
Environmental Research and Public Health

MDPI

File S2

Second Database Search Strategy
Search Date: 11/4/22 to 05/31/24
Before De-duplication
CINAHL Plus: 4
Health Source: Nursing/Academic Edition: 5
Nursing & Allied Health Database: 0
Medline via PubMed: 15
SPORTDiscus: 0
Web of Science: 0
Citation Total: 24

After De-Duplication
CINAHL Plus: 4
Health Source: Nursing/Academic Edition: 5
Nursing & Allied Health Database: 0
Medline via PubMed: 14
SPORTDiscus: 0
Web of Science: 0
Citation Total: 23

CINAHL Plus (1985-2024)

Line	Search Term	Hits
S1	(MH "Aging") OR (MH "Aged, 80 and Over") OR (MH "Age Factors") OR (MH "Geriatricians") OR (MH "Frailty Syndrome")	490,467
S2	elder* OR geriatric* OR ((old* or aged) N1 (person* OR adult* OR people OR patient*)) OR frail OR senior*	338,192
S3	S1 OR S2	708,944
S4	(MH "Head Injuries") OR (MH "Contusions and Abrasions") OR (MH "Brain Injuries") OR (MH "Brain Concussion") OR (MH "Brain Contusions")	40,712
S5	Contusion OR concussion OR head injury OR mTBI OR "mild traumatic brain injury"	27,757
S6	S4 OR S5	50,380
S7	(MH "Fall Risk Assessment Tool") OR (MH "Risk Assessment") OR (MH "Risk Factors")	314,306
S8	Fall risk	11,355
S9	S7 OR S8	322,272
S10	(MH "Accidental Falls")	25,822
S11	Fall*	81,058
S12	S10 OR S11	81,058
S13	MH "Prospective Studies"	512,638
S14	(prospective or follow up)	779,150
S15	S13 OR S14	779,150
S16	S3 AND S6 AND S9 AND S12 AND S15	39
S17	Filter for Publication Date 20221101-20240531	
S18	Filter for English	39

Health Source: Nursing/Academic Edition (1946-2024)

Line	Search Term	Hits
S1	XX "aging" OR XX "geriatrician" OR XX "geriatric" OR XX "adult"	150,994
S2	TX “aged 80 and over” OR TX “frailty syndrome” OR TX “age factors” OR TX “frail” OR TX “elderly” OR TX “aged” OR TX “older” OR TX “elder” OR TX “geriatric” OR TX “elderly people” OR TX “old people” OR TX “senior”	543,630
S3	S1 OR S2	623,354
S4	XX “contusions” OR XX “abrasion” OR XX “concussion” OR XX “TBI”	5,928
S5	TX “head injury” OR TX “head injuries” OR TX “brain injuries” OR TX “brain injury” OR TX “brain injuries” OR TX “mTBI” OR TX “mild traumatic brain injury”	26,844
S6	S4 OR S5	28,301
S7	XX “risk factors”	109,594
S8	TX “fall risk” OR TX “fall risk assessment tool” OR TX “risk assessment”	48,021
S9	S7 OR S8	145,263
S10	XX “accidental injury”	177
S11	TX “accidental falls” OR TX “fall”	116,198
S12	S10 OR S11	116,358
S13	DE "longitudinal method"	43,797
S14	S3 AND S6	11,331
S15	S14 AND S9	1,524
S16	S15 AND S12	704
S17	S16 AND S13	21
S19	Filter for Publication Date: 20221101-20240531	
S19	Filter for English	21

International Journal of
Environmental Research and Public Health
MDPI

Nursing and Allied Health Database (1857-2024)

Line	Search Term	Hits
S1	mesh.Exact("Aging" OR "geriatrician" OR "geriatric" OR "adult")	224,651
S2	"aged 80 and over" OR "frailty syndrome" OR "age factors" OR "frail" OR "elderly" OR "aged" OR "older" OR "elder" OR "geriatric" OR "elderly people" OR "old people" OR "senior"	1,582,534
S3	S1 OR S2	1,637,273
S4	mesh.Exact("contusions" OR "abrasion" OR "concussion" OR "TBI")	165
S5	"head injury" OR "head injuries" OR "brain injuries" OR "brain injury" OR "brain injuries" OR "mTBI" OR "mild traumatic brain injury"	89,737
S6	S4 OR S5	89,869
S7	mesh.Exact("risk factors")	54,880
S8	"fall risk" OR "fall risk assessment tool" OR "risk assessment"	170,591
S9	S7 OR S8	215,303
S10	mesh.Exact("accidental falls")	2,227
S11	"accidental falls" OR "fall"	359,468
S12	S10 OR S11	359,468
S13	mesh.Exact("prospective studies" OR "Follow-Up Studies")	62,533
S14	S3 AND S6	48,894
S15	S14 AND S9	4,497
S16	S15 AND S12	1,996
S17	S16 AND S13	77
S18	Filter for Publication Date: 2022-11-04 to 2024-05-04	
S19	Filter for English	77

Medline via PubMed (1879-2024)

Line	Search Term	Hits
S1	"Aged"[Mesh] OR "Aged, 80 and over"[Mesh] OR "Aging, Premature"[Mesh] OR "Healthy Aging"[Mesh] OR "Aging"[Mesh] OR "Cognitive Aging"[Mesh] OR "Geriatrics"[Mesh] OR "Frail Elderly"[Mesh] OR "Long-Term Care"[Mesh] OR "Homes for the Aged"[Mesh]	3,643,246
S2	"geriatric care" OR "elder*" OR "old" OR "older" OR "geriatric*" OR "age" OR "aged" OR "people*" OR "patient*" OR "frail*"	12,492,132
S3	S1 OR S2	12,569,239
S4	"Head Injuries, Closed"[Mesh] OR "Head Injuries, Penetrating"[Mesh] OR "Brain Contusion"[Mesh] OR "Brain Injuries"[Mesh] OR "Brain Concussion"[Mesh]	83,190
S5	"abrasions" OR "concussion" OR "head injury" OR "mTBI"	36,971
S6	S4 OR S5	100,565
S7	"Risk Factors"[Mesh] OR "Accident Prevention"[Mesh] OR "Uncertainty"[Mesh] OR "Risk Assessment"[Mesh] OR "Geriatric Assessment"[Mesh]	1,272,069
S8	"prevention*" OR "risk*" OR "protective*" OR "assess*" OR "factor*"	11,832,800
S9	S7 OR S8	11,871,240
S10	"Accidental Falls"[Mesh]	27,654
S11	"fall*"	310,344
S12	S10 OR S11	310,344
S13	"Prospective Studies"[Mesh]	641,892
S14	"Follow-Up Studies"[Mesh]	688,076
S15	S13 OR S14	1,237,170
S16	S3 AND S6	59,137
S17	S16 AND S9	33,362
S18	S17 AND S12	1,767
S19	S18 AND S15	238
S20	Filter Publication Date: November 2022 to May 2024	
S21	Filter for English	225

International Journal of
Environmental Research and Public Health

MDPI

SPORTDiscus with Full Text (1573-2024)

Line	Search Term	Hits
S1	(DE "AGING") OR (DE "GERIATRICS")	7,880
S2	TX (“aged 80 and over” OR “adult” OR “frailty syndrome” OR “age factors” OR “frail” OR “elderly” OR “aged” OR “older” OR “elder” OR “geriatrician” OR “geriatric” OR “elderly people” OR “old people” OR “senior”)	381,434
S3	S1 OR S2	384,013
S4	(DE "BRUISES") OR (DE "BRAIN concussion") OR (DE "BRAIN injuries")	6,737
S5	TX (“contusion” OR “abrasion” OR “head injury” OR “head injuries” OR “brain injuries” OR “brain injury” OR “brain injuries” OR “mTBI” OR “mild traumatic brain injury”)	24,537
S6	S4 OR S5	26,124
S7	DE "INJURY risk factors"	1,928
S8	TX (“fall risk” OR “fall risk assessment tool” OR “risk assessment”)	17,206
S9	S7 OR S8	18,953
S10	DE "ACCIDENTAL falls"	1,865
S11	TX (“accidental falls” OR “fall”)	135,647
S12	S10 OR S11	135,647
S13	TX (“prospective studies” OR “Follow-Up Studies”)	11,327
S14	S3 AND S6	10,444
S15	S14 AND S9	620
S16	S15 AND S12	336
S17	S16 AND S13	47
S18	Filter Publication Date: 20221101-20240531	
S19	Filter for English	47

Web of Science (Web of Science Citation Index Expanded, 1977-2024)		
**Line**	**Search Term**	**Hits**
S1	ALL=("aging" OR "geriatrics" OR "aged 80 and over" OR "adult" OR "frailty syndrome" OR "age factors" OR "frail" OR "elderly" OR "aged" OR "older" OR "elder" OR "geriatrician" OR "geriatric" OR "elderly people" OR "old people" OR "senior")	2,935,604
S2	ALL=("Head Injuries, Closed" OR "Head Injuries, Penetrating" OR "Brain Contusion" OR "Brain Injuries" OR "Brain Concussion" OR "bruises" OR "contusion" OR "abrasion" OR "head injury" OR "head injuries" OR "brain injuries" OR "brain injury" OR "brain injuries" OR "mTBI" OR "mild traumatic brain injury")	181,275
S3	ALL=("risk factors" OR "fall risk" OR "fall risk assessment tool" OR "risk assessment")	976,518
S4	ALL=("accidental falls" OR "fall")	233,601
S5	ALL=("prospective studies" OR "follow-up studies")	59,675
S6	S1 AND S2	28,159
S7	S6 AND S3	2,072
S8	S7 AND S4	179
S9	S8 AND S5	1
S10	Filter by Publication Years: 2022, 2023, 2024	0
S11	Filter for English	0
